# Health Care Providers’ Perspectives of Clinical Decision Support Tools for Pediatric Sepsis in Bangladesh: Qualitative Study

**DOI:** 10.2196/73451

**Published:** 2025-09-26

**Authors:** Shamsun N Shaima, Alicia E Genisca, Md Tanveer Faruk, Md Fakhar Uddin, Akash Saha, Nadia Sultana, Nidhi Kadakia, Monique Gainey, Elleen Kim, Kikuyo Shaw, Farzana Afroze, Joan Chepngeno, Atin Jindal, Sifat A Chowdhury, Md Jobayer Chisti, Adam C Levine, Stephanie C Garbern

**Affiliations:** 1 International Centre for Diarrhoeal Disease Research Dhaka Bangladesh; 2 Department of Nutritional Sciences School of Graduate Studies University of Toronto Toronto, ON Canada; 3 Department of Emergency Medicine Warren Alpert Medical School of Brown University Providence, RI United States; 4 Department of Pediatrics Warren Alpert Medical School of Brown University Providence, RI United States; 5 Department of Epidemiology and Community Health University of North Carolina at Charlotte Charlotte, NC United States; 6 Department of Emergency Medicine Yale School of Medicine New Haven, CT United States; 7 Virginia Tech Carilion School of Medicine Roanoke, VA United States; 8 Warren Alpert Medical School of Brown University Providence, RI United States; 9 Brown University Providence, RI United States; 10 Department of Internal Medicine Warren Alpert Medical School of Brown University Providence, RI United States; 11 Division of Biology and Medicine Brown University Providence, RI United States

**Keywords:** global health, sepsis, pediatrics, digital health, implementation science, mobile health

## Abstract

**Background:**

Sepsis, a life-threatening condition resulting from a dysregulated immune response to infection, disproportionately affects children in low- and middle-income countries (LMICs). Children with sepsis in LMICs face high mortality rates, with early detection and clinical monitoring posing significant challenges to effective management. There is great potential for digital technologies, such as wearable biosensor devices and mobile health (mHealth) clinical decision support (CDS) tools, together referred to as clinical decision support systems (CDSSs), to enable closer monitoring and more prompt recognition of children at risk of advanced sepsis and death. However, little is known about the perceptions of health care providers (HCPs) regarding the introduction of new digital health tools for pediatric sepsis care in LMICs.

**Objective:**

The objective of this study was to assess HCPs’ understanding, perceptions, and recommendations regarding the design and implementation of digital CDSSs for pediatric sepsis care in Bangladesh.

**Methods:**

Between February and May 2024, 18 individual semistructured in-depth interviews were conducted with HCPs (nurses and physicians) at 3 urban hospitals in Bangladesh. The data were transcribed, translated from Bangla to English, and analyzed using a framework matrix analysis approach. Participants were asked about familiarity with digital health tools, feedback on CDSS design, perceptions of the system’s utility, and barriers and facilitators to use of similar tools in clinical settings in Bangladesh.

**Results:**

Participants reported overall positive perceptions toward the potential implementation of a CDSS for pediatric sepsis care in Bangladesh. Some key priorities for the design of a CDSS were durability, reusability, cost considerations, reliability, and accuracy. Clinicians desired the CDS tool to also have customizable alarm parameters and include additional functions such as glucose monitoring. Many favored audio (ringtone) or visual (light) alarms to alert about changes in captured vital signs. HCPs believed that a CDSS could enhance patient care by allowing greater staff capacity to monitor patients, reducing management time, and aiding in faster clinical decision-making, with some suggesting it could lower mortality rates. Concerns regarding implementation included internet availability, affordability of the wearable devices, and trust in the CDSS outputs compared to expert clinician judgement.

**Conclusions:**

The findings of this study highlight HCPs’ perceptions toward the potential of wearable biosensor devices and CDS tools (CDSSs) for improving pediatric sepsis outcomes in LMICs and highlight the need to address implementation challenges to ensure the effective integration of CDSSs into health care systems.

## Introduction

Sepsis is a life-threatening condition characterized by a dysregulated immune response to infection, which can lead to death if not promptly treated [1]. It is one of the leading causes of child mortality worldwide, with more than half of all sepsis cases worldwide occurring in children younger than 5 years [2,3], and remains a significant global health concern [4]. As of 2017, there were an estimated 2.9 million sepsis-related deaths among children younger than 5 years [5]. The burden of sepsis disproportionately affects children in low- and middle-income countries (LMICs) for a multitude of reasons, including lack of human, infrastructural, and material resources necessary to adhere to the current best practice protocols, which are usually based on standards developed in high-income countries.

The management of potentially septic children is challenging, as many children with infections do not progress to having sepsis, while those with sepsis can rapidly and unexpectedly decompensate, developing shock or multiorgan failure. The ability to provide close monitoring and risk stratify septic children is often only available in higher-resourced emergency and critical care wards in referral or specialized pediatric care facilities in LMICs [2,3]. Early anticipation of poor outcomes, including shock, is a mainstay of managing sepsis, as rapid treatment initiation significantly improves outcomes and can prevent or minimize the severity of multiple organ failure [3,4]. In high-income countries, diagnostic and prognostic tools, continuous monitoring systems, and advanced resources for resuscitation are typically recommended to optimize care. However, in LMICs, the feasibility of implementing recommended measures may be limited due to a lack of such resources, increasing the risk of severe complications [3,6]. In Bangladesh, a lower middle–income South Asian country, sepsis remains the leading cause of childhood mortality [7].

Digital health technologies, including wearable biosensor devices, artificial intelligence, and mobile health (mHealth) clinical decision support (CDS) tools, have great potential to mitigate the lack of critical care resources in LMICs by enabling closer monitoring of vital signs, allowing for more prompt recognition of children at risk of advanced sepsis and death [8]. Given the lack of consensus in pediatric sepsis care protocols, CDS tools could be beneficial in streamlining clinical knowledge and patient care. Current developments are addressing this challenge of defining optimal sepsis criteria and promoting its facilitation through CDS tools [9,10]. Recent studies have also demonstrated the ongoing evolution of CDS tools, which may now incorporate AI-driven algorithms and predictive models based on real-time physiological data, rather than solely relying on laboratory diagnoses [11,12]. These advancements further enhance the ability of CDS tools to detect and risk stratify patients with sepsis, enabling clinicians to provide care more promptly [13,14]. It is also recognized that existing tools for sepsis management are often designed for use by highly trained health care providers (HCPs) or rely on infrastructure, such as electronic health records, to function. However, in LMICs, clinicians have varying levels of pediatric sepsis awareness, infrastructural availability, and digital literacy [15]. The adaptation of new tools for sepsis care in LMICs therefore necessitates customization to fit various context-specific factors [10].

Understanding the perceptions of HCPs regarding digital health and CDS tools for pediatric sepsis care in LMICs is critical to ensure the successful implementation and adoption of such technologies and realize their immense potential [16-18]. This study aimed to assess HCPs’ understanding and perceptions of digital and CDS tools for pediatric sepsis care in LMICs, in the context of the development of a novel clinical decision support system (CDSS) for pediatric sepsis care in Bangladesh.

## Methods

### Study Setting, Population, and Design

This study was conducted as part of a larger study called REMEDIES (Remote Electronic Monitoring and Decision Support for Improved Sepsis Care), which aims to develop a novel, wearable device-enabled mHealth CDSS for sepsis care in Bangladesh. The CDSS consists of a wearable biosensor device that collects real-time vital sign information and is linked to an mHealth CDS tool that can report vital signs and use machine learning algorithms to predict sepsis [19]. Questions were included to gain feedback on a prototype version of this CDSS, as well as on digital technology more generally.

**Figure 1:**
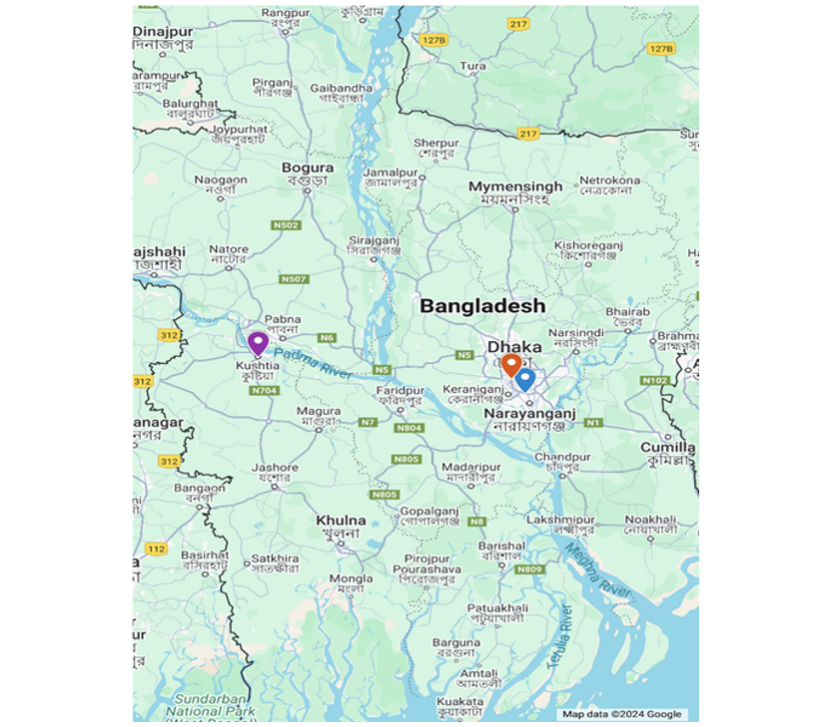
Study site locations. The purple icon represents Kushtia General Hospital; orange represents the International Centre for Diarrhoeal Disease Research, Bangladesh; and blue represents the Institute of Child and Mother Health.

Eighteen semistructured in-depth interviews were conducted in person with HCPs at 3 hospitals in urban Bangladesh from February to May 2024. The inclusion criterion was being an HCP who had at least 1 year of experience providing care for pediatric patients with sepsis at 1 of the 3 study sites. Purposive selection was used to select potential participants for inclusion, ensuring diversity of clinical experience. The only exclusion criterion was declining to participate in the study. Three nurses and 3 physicians were interviewed at each of the 3 sites. Interviews were conducted in the local language, Bangla, at the participants’ respective hospitals. The hospitals included were the International Centre for Diarrhoeal Disease Research, Bangladesh (icddr,b) Dhaka Hospital in Dhaka, Kushtia General Hospital (KGH) in Kushtia, and Institute of Child and Mother Health (ICMH) in Matuail (Figure 1). The icddr,b Dhaka Hospital is a private, nonprofit research hospital in Bangladesh’s capital city, which treats about 200,000 patients yearly, over half of whom are pediatric patients younger than 5 years. The hospital has a team of 42 doctors, 82 nurses, and 150 support staff [20]. KGH is a 250-bed government hospital that provides care to approximately 83,000 patients annually, 16% of whom are younger than 5 years. ICMH in Dhaka is a maternal and neonatal specialty care hospital, with over 90 beds dedicated to the pediatric and neonatal special care units. It is one of the largest Special Care Newborn Units in Bangladesh [21].

The SRQR (Standards for Reporting Qualitative Research) checklist is provided in Multimedia Appendix 1.

### Study Training

The primary qualitative research team consisted of 2 physicians with pediatric and critical care experience and 2 anthropologists, who participated in a collaborative qualitative research theory and methodology training led by study investigators from icddr,b and Brown University from November 2023 until January 2024. Training included a 9-part lecture series on qualitative research as well as a practical component in which interviewers and the notetaker conducted mock interviews with other members of the research staff to provide feedback on the interviewer’s tone, provide alternative methods for asking questions, and prepare interviewers to troubleshoot typical challenges faced when conducting interviews. The first 2 participant interviews were conducted with other research staff observing in order to provide feedback after the interview concluded.

### Data Collection

The interviews were designed to explore HCPs’ experiences and challenges with pediatric sepsis management; awareness and perceptions of digital health tools, with a focus on their potential use in monitoring and treating pediatric sepsis; and recommendations and preferences regarding future implementation in Bangladesh and similar settings. Participant demographic information (eg, age and years of practice) was collected via a participant demographic questionnaire (Multimedia Appendix 2).

**Figure 2:**
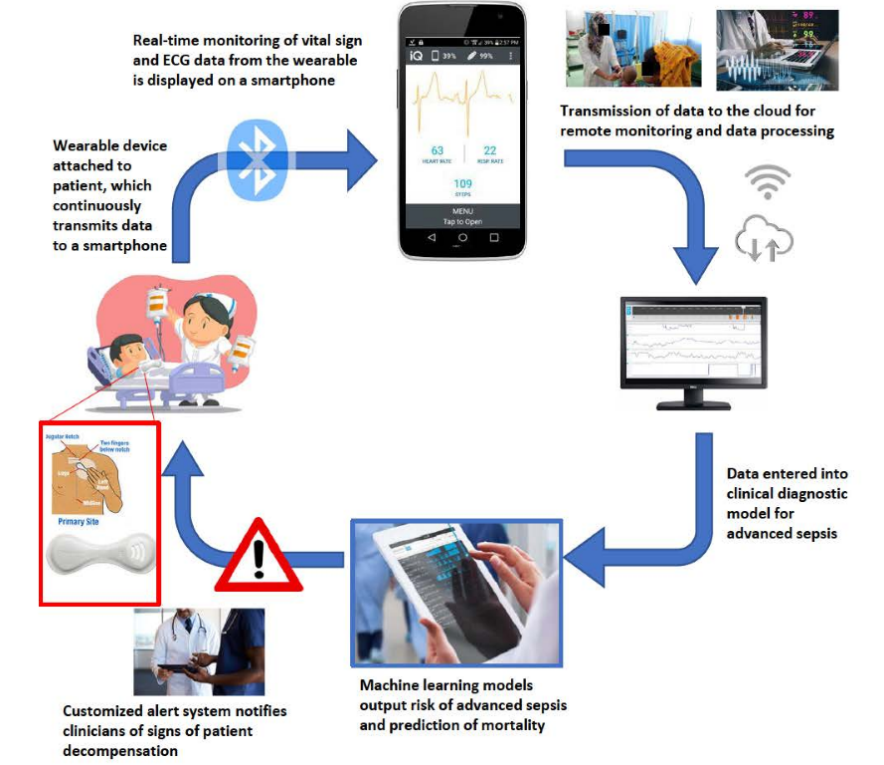
Flow schematic of the clinical decision support system. ECG: electrocardiography.

A comprehensive semistructured interview guide (Multimedia Appendix 3) was created to gather feedback on the development of the CDSS for pediatric sepsis care in Bangladesh. The guide consisted of several key sections, including participants’ familiarity with digital tools, such as wearable biosensor devices and CDS tools, in both personal and professional contexts. During the interviews, participants were shown a wearable biosensor device prototype that linked to a prototype mHealth CDS tool, which captured live vital sign information. This was demonstrated to the participants using either the interviewer or a designated mannequin as a model. This allowed the participants to observe the real-time functionality of the wearable biosensor device and mHealth CDS tool displayed on a mobile device (Figure 2).

Data were then collected on the provider’s opinions of the CDSS, including its potential benefits, challenges, considerations for implementation, and specific features and functionalities participants would find valuable, such as alert mechanisms and CDS capabilities. Each interview was conducted by a team of research staff and clinicians from icddr,b, who were fluent in the local language (Bangla) and English, and a notetaker was present at each interview to facilitate and clarify questions. The sessions were audio-recorded, transcribed, and translated into English for analysis. To mitigate participant fatigue, individuals were informed that they could take breaks at any time during the session, and culturally appropriate snacks were available to support engagement throughout the interview process. Of the 18 interviews, only 3 ran longer than the proposed 90 minutes.

Debrief forms were completed by the interviewers and the notetaker after each session to summarize key points and assess data saturation. Data saturation was validated through a team-based consensus. Prior work has shown that data saturation can be satisfactorily obtained with 9-17 interviews [22], and our work analyzed 18 interviews. Data quality was assured by reviewing all English transcripts for clarity of meaning with the interviewer team.

### Data Analysis

Data were analyzed using a framework matrix approach. A framework matrix is a systematic method for qualitative data analysis that organizes data into a matrix of rows (cases) and columns (codes), allowing researchers to compare and contrast data across and within cases while preserving the context of each participant’s views [23].

Six framework themes were analyzed: (1) familiarity with digital health tools and devices, (2) perceptions about CDSS use for patient monitoring among children with sepsis, (3) perceptions about CDSS use for clinical decision-making among children with sepsis, (4) barriers and facilitators to the adoption of a CDSS, (5) feasibility, and (6) CDSS functionality.

Each theme was further split into codes corresponding to individual interview questions, and each participant was assigned to a row. The research team had primary and secondary coders (7 in total) to complete the framework matrices and provide summaries for each. Frameworks were double coded in this way to flag discrepancies between coders, followed by the resolution of any disagreements through consensus to enhance credibility and dependability. This iterative and collaborative process helped ensure consistency and reduced individual bias in the analysis. Our team maintained a detailed audit trail documenting analytic discussions throughout the study for confirmability and validity. We provide descriptions of participant experiences and health care settings to allow assessment for applicability to similar clinical contexts.

The multidisciplinary research team, which included Bangladesh- and US-based physicians, research assistants, and anthropologists, engaged in ongoing reflexivity to recognize and balance the varied positionalities while interpreting the experiences of the Bangladeshi physician and nurse participants.

Basic descriptive statistics were used to calculate frequencies, percentages, medians, and IQRs.

### Ethical Considerations

Ethical approval for this project was obtained from the icddr,b Institutional Review Board (IRB) (including the Research Review Committee and Ethical Review Committee) and the Rhode Island Hospital IRB. Participants were informed that personal data would not be recorded or stored. Each participant was assigned a study ID based on study location and their role. Transcribed and translated data from the participants were always stored in a password-protected folder on a secure study computer. Written consent in Bangla or English was obtained after participants were allowed to ask questions. Participants from KGH and ICMH were offered a monetary honorarium in Bangladeshi taka (1600 [US $13.14] for physicians and 800 [US $6.57] for nurses) for their participation in the study. Participants from icddr,b, who were not permitted to receive a monetary honorarium, received gift items such as a diary and pen.

## Results

### Participant Demographics

A total of 18 participants (9 nurses and 9 physicians) were invited to take part in the study, and 6 interviews were conducted at each facility. The majority of participants were female and aged 25-34 years. The median number of years of experience treating sepsis was 7 years. The demographic characteristics of the participants are shown in Table 1. Each interview lasted between 50 and 100 minutes.

**Table 1:** Demographic characteristics of the participants.

Characteristic		Value (N=18)
**Age (years), n (%)**		
	18-24	1 (6.0)
	25-34	9 (50.0)
	35-44	4 (22.2)
	45-54	4 (22.2)
**Gender, n (%)**		
	Male	5 (27.8)
	Female	13 (72.2)
**Education, n (%)**		
	Nursing diploma	9 (50.0)
	Bachelor of Medicine, Bachelor of Surgery (MBBS) degree	9 (50.0)
	Master of Public Health (MPH) degree	7 (38.9)
**Working status, n (%)**		
	Part time	1 (5.6)
	Full time	17 (94.4)
**Current position, n (%)**		
	Nurse	9 (50.0)
	Doctor	9 (50.0)
**Approximate number of children with sepsis treated per month, n (%)**		
	2-5	5 (27.8)
	6-10	4 (22.2)
	11-20	4 (22.2)
	>20	5 (27.8)
**Experience, median (IQR)**		
	Years at the current position	8.5 (6-11)
	Years treating children with sepsis	7 (5-9.5)

### Familiarity With Digital Health Tools and Devices

The majority of respondents reported familiarity with a range of digital devices, including smartphones, tablets, laptops, wearable devices (eg, smartwatches), and desktop computers. Additionally, most respondents reported being familiar with digital health devices used in health care workplaces, such as hospital computers, smartphones, ultrasound machines, and cardiac monitors, and digital tools for patient assessment, such as digital thermometers, scales, oximeters, glucometers, ventilators, and blood pressure monitors. However, some respondents also understood “digital” to include electronic devices that were not always classified as digital devices, such as infusion pumps, mechanical ventilators, and infant warmers.

At work we have cardiac monitors, infusion pumps, syringe pumps, digital thermometers, pulse oximeters and many more.

Nurse from icddr,b

I have to use different kinds of digitized machines in the hospital. First of all, when the babies come, we take the weight at first on [the] weight machine, which is digital. Then, we use [a] digital thermometer, digital glucometer, [and] check the saturation [with a] pulse oximeter, and we have a cardiac monitor through which we attach the baby with the leads.

Physician from icddr,b

Then there are devices. I use syringe pump, CPAP, then mechanical ventilator. Also warmer is one kind of device. These are the maximally used.

Physician from ICMH

When asked specifically about their familiarity with wearable biosensor devices, HCPs commonly identified pulse oximeters and traditional (ie, wired) bedside cardiac monitors as wearable digital devices used in clinical settings. Personal use or general awareness of smartwatches was also prevalent, although these were not mentioned in clinical use in health care settings. Awareness of wireless, wearable biosensor devices was mentioned by members of icddr,b in a prior phase of this study, although this was not mentioned by participants from the other study sites. Overall, although a notable number of participants expressed a lack of familiarity with wearable biosensor technology in health care settings, the idea of a wearable biosensor device for monitoring vitals for clinical care was readily understood after a brief demonstration and was overall received positively.

Yes, just like the pulse oximeter [it] can be applied to hands and feet. Currently we use a pulse oximeter, something like this. It looks like you put it on your arm and leg. I mean, something like a pulse oximeter that would record my totals (vitals), that would be great.

Nurse from KGH

### Perceptions About CDSS Use for Patient Monitoring Among Children With Sepsis

In terms of using a digital device for monitoring children with sepsis, overall, both nurses and physicians felt that a wearable biosensor device would be helpful in providing continuous vital sign monitoring. Several providers explained that by relying less on manual vital sign assessment, they would be able to provide more efficient care and the workload on health care workers could be decreased, especially during times of high patient load and personnel shortage. More importantly, wireless wearable biosensor devices were identified as a way to minimize patient discomfort related to monitoring, especially in children.

Non-invasive alternative [and] minimizing discomfort for children. Just like how pulse oximeters revolutionized oxygen level monitoring non-invasively, future innovations might similarly advance capabilities like blood gas or glucose monitoring, potentially transforming clinical practice.

Physician from ICMH

Furthermore, both physicians and nurses at each of the clinical sites emphasized their perception that the CDSS should be used primarily for monitoring purposes, and making a diagnosis (eg, for sepsis) would require additional information.

Diagnosis will require everything starting from history. Here it only seems like monitoring. In terms of monitoring, it will be very good, but there is much more in the diagnosis area that seems not dependent on the device.

Physician from KGH

Several nurses mentioned that, as a continuous, remote monitoring device, the CDSS would allow them to efficiently communicate a patient’s status better by facilitating the early detection of deteriorating patients and prompting them to notify their physicians more rapidly. Many participants also emphasized the benefit of this type of technology over the current standard of care for monitoring in their workplaces.

This would enable us to inform the doctor promptly, and the doctor could also monitor the data. As nurses, we would be able to provide special care to these babies.

Nurse from ICMH

If the monitoring system is like this, we can do a lot of good with it.

Nurse from KGH

Additionally, many felt the CDSS would provide reassurance to the caretakers or families of patients, as the caretaker would know their child was being continuously monitored, especially in lower-resourced hospitals, where caretakers are often relied upon to notify staff of changes in the clinical condition.

Yes, it would be beneficial for doctors and nurses to counsel the patient's guardians, reassuring them that the monitoring machine is always connected to their child. This would provide peace of mind, knowing that medical staff are actively monitoring their condition. While ultimate outcomes are in Allah's hands, constant monitoring can alleviate mental distress for families.

Nurse from ICMH

### Perceptions About CDSS Use for Clinical Decision-Making Among Children With Sepsis

Both groups of providers expressed their belief that the overall quality of care would be improved by digital devices, allowing them to identify sepsis earlier and promptly take appropriate action (eg, adjusting the course of treatment in response to information from digital tools).

In all likelihood, if this device is in front of me, it won't take more than a minute to complete everything by monitoring vital signs because every minute counts for sepsis.

Physician from icddr,b

It saves me time, and I can give that time to another patient.

Nurse from icddr,b

I can make an early diagnosis that the child has sepsis and start early sepsis management.

Physician from icddr,b

After demonstrating an example of a wearable biosensor device and mobile CDS tool, the providers across all hospital systems further detailed their perspectives on how such a CDSS would be used to make clinical decisions. Nurses focused more on how quicker notification of abnormal vitals will guide them to seek assistance from the physician, while physicians discussed how such a system can be used to prioritize more severe patients, facilitate early treatment initiation, and assist with referral decisions. Several physicians also highlighted the versatility of the wearable biosensor device, explaining how the device’s vital outputs can also be used for clinical vital sign monitoring in other diseases besides sepsis.

As it is a continuous vital monitoring device, vitals are coming in. If I can do early identification then my patient care, which means patient categorization, will be easy. A patient with an abnormality in [their] vitals will be categorized beforehand. So, in that case, we will be able to identify sepsis in advance if it is going from sepsis to severe sepsis. We can identify first seeing those vitals. So, if we get the information from a single device from the individual patches of each patient, that's our early identification.

Physician from icddr,b

I can see that the patient is fine or deteriorating, and I can take action immediately.

Physician from KGH

I should prioritize that patient. There may be more opportunities for early intervention...I can ensure early intervention.

Physician from ICMH

Compared to providers at icddr,b, those at other sites, specifically physicians, seemed more cautious about the CDSS’s implementation and indicated that providers should not over-rely on digital devices as they cannot replace clinical gestalt.

The number one caution to be taken is primarily [to] never trust a machine over a human. Most importantly, it is most important that my clinical knowledge and expertise is not a substitute or its replacement because the critical patient needs to be judged by my own clinical eye. Overemphasis [on] the device can harm someone's condition.

Physician from ICMH

Diagnosis will require everything starting from history. Here it only seems like monitoring. In terms of monitoring, it will be very good, but there is much more in the diagnosis area that seems not dependent on the device.

Physician from KGH

### CDSS Use for Risk Stratification and Clinical Prediction

Respondents reported that the function of the CDSS to provide risk estimates of sepsis severity or mortality risk would help to quickly identify patients at the highest risk of death, inform family discussions regarding the patient’s prognosis, and help clinicians allocate resources.

Being able to predict these conditions (MODS, shock) in advance is crucial. By predicting them early, we can monitor the likelihood of death in advance and prepare accordingly.

Physician from ICMH

If I can pick the deterioration very early, my mortality rate will be much lower. The deterioration will be reduced, I will be able to shift the child's treatment plan very soon and go to high management. The children will be saved faster.

Physician from icddr,b

There also appeared to be discrepancies in the understanding of the different functions of the device in terms of both monitoring and risk stratification. Some respondents, when asked specifically to give feedback about risk estimate predictions, appeared to understand this function as the same as monitoring. Additionally, some respondents reported doubts about whether they could trust the risk estimates provided by the CDSS.

If there is a risk of death, after looking at the tool, I have to [think] myself. It is not possible for me to completely trust the device alone for the patient.

Physician from ICMH

### Barriers and Facilitators to the Implementation of a CDSS

#### Barriers

The key barriers to the adoption of a CDSS identified by participants included the following: need for specific training on how to use the devices, perceived high device cost, device maintenance challenges, high patient volume, limited personnel, and concerns about reliability.

Both nurses and physicians emphasized the need for proper training, infrastructure, and device availability and maintenance to successfully use the device in their respective workplace settings. Several highlighted the importance of first educating health workers on the advantages of the devices, suggesting that a greater understanding of the benefits of the device was important to support increased uptake, given a general lack of awareness of newer digital technologies in their workplaces.

At first, keep in mind that the doctors and nurses should be sensitized about the device. If they are aware of the perks of using this device, then they will definitely concentrate on using this device and have the best use of it.

Physician from KGH

The cost associated with the CDSS, including both initial purchase and ongoing maintenance, and whether additional financial burden would be placed on patients and their families, emerged as a prominent barrier. Several providers, noting that their hospital primarily serves low-income patients, expressed worries about whether the costs of the devices would be borne by the hospital or passed on to families, potentially adding financial strain. The importance of a realistic and sustainable financial model to ensure long-term uptake and use of these technologies was also stressed by participants.

Often, if the cost is high, the family cannot afford it. Some patients are too poor to afford NICU admission treatment. So, how will the cost of the machine be covered?

Nurse from ICMH

If the cost were lower and there was a system in place to provide more devices to the hospital, it would be beneficial for both us and the children. We could ensure that all children with sepsis receive one, allowing us to monitor their vital signs continuously.

Nurse from ICMH

It must be affordable because it is a government hospital where very poor people come to get services, [people] who think they don’t have the capacity to get services outside.

Nurse from KGH

Most providers emphasized that training would be essential for both physicians and nurses before implementing new digital tools, particularly in terms of understanding how to properly handle and use the devices. Some respondents suggested that training a few individuals in advance, who could then train the rest of the staff (ie, train-the-trainer approach), would be the most effective approach. Others felt that simultaneous profession-specific training for both nurses and physicians would be more beneficial.

If everyone can use it, understand it properly or understand the monitoring properly, if everyone learns it or slowly gets used to it, ultimately the workload on me will be reduced. The workload on other doctors or colleagues who will come in my place or who are in other shifts will also be reduced. When the workload on a doctor is less, the quality of work improves.

Physician from icddr,b

If our seniors can provide us with sufficient training, then I believe it will definitely be helpful. We also have our sisters [nurses] here, and doctors as well, so if everyone can be trained, then it will be beneficial.

Physician from icddr,b

High patient load and limitations in terms of manpower were barriers identified by both physicians and nurses across the different hospitals. The providers reported that pediatric wards usually have a high patient volume and are busy, and therefore, managing and tracking devices could be challenging. One of the physicians noted that if manpower issues could be addressed, using digital devices would be feasible.

I would say this facility is patient based. We have uncountable patients here. Whereas our capacity in pediatric ward is 20 and in SCANU [is] 11. In that place, we always have more than 20 patients in our facility. This is the main barrier of using devices in our facility. In case of manpower, we have enough sisters [nurses]. We lack doctors, and I think that can be overcome, and thus digital device can be used. No problem.

Physician from KGH

In addition, there were concerns about whether the wearable biosensor devices could be reused for multiple patients and their risk of causing nosocomial infection.

I think it's better not to transfer the device from one patient to another because it was attached to their body. Doing this could increase the risk of spreading infections, especially since children have weaker immune systems. Reusing the device could spread germs, so it’s best to use one device per child.

Nurse from ICMH

Many respondents discussed that maintenance of the devices could be a potential challenge, particularly for devices that require charging. A nurse from icddr,b shared that wearable biosensor devices might be prone to damage or contamination, for example, from vomit and stool, as children with sepsis and infectious diseases often experience these symptoms.

In icddr,b, I don’t think there will be any constraints. But since it is a diarrheal hospital, there’s a possibility that the device [will] get ruined with puke and stool. It can be a problem since the kids tend to throw up.

Nurse from icddr,b

Respondents also identified several barriers related to perceptions of a CDSS, including negative attitudes, resistance to change, a lack of trust in the new technology, and difficulties in integrating the device into daily clinical practice. One of the providers explained that acceptance of new devices could be a challenge, as providers are accustomed to traditional methods, and adapting to new technologies takes time. A few providers also felt that new tools would not be as reliable as their current standard of care and would necessitate that providers verify their accuracy.

If we are introduced with a new device, first of all, my challenge will be how to use that?! I will face difficulty about if I am properly using the tool and if I am understanding the indications of the tool. I mean the total use of the device and understanding the whole use of the tool will take some time at first. Second, acceptance. We are habituated with old things, and hence we take some time to accept new things.

Physician from icddr,b

Providers expressed concerns about the sustainability of the implementation of a CDSS, particularly once the research project support ends. They felt that, without follow-up or ongoing maintenance, devices may stop functioning properly over time. It was also emphasized that a lack of coordination and administrative commitment to maintenance and quality improvement could hinder the long-term use and effectiveness of a CDSS.

This lack of coordination, the administrative sincerity [commitment] to improve the quality of work is a serious problem. If we can't overcome it, then [why you are] spending a lot of money on …studies, I don't understand, if they can't benefit the people!

Physician from KGH

It will cost money. They [hospitals] have no money to buy medicine; how will they buy it? They may agree with the government. But when it ends later, it will cause problems. It will cause problems with quality; it will cause problems with availability.

Physician from KGH

#### Facilitators

Identified facilitators to the use of a CDSS for the management of children with sepsis were ease of integration with existing patient monitoring systems, ease of use, availability of infrastructure including electricity and reliable Wi-Fi, staff readiness, and perceived benefits compared to the current standard of care. One provider highlighted that the resources they were currently using to manage sepsis were not enough and that there was a need for better tools and technology. A nurse at ICMH added that the availability of phone chargers at each bed, along with Wi-Fi access throughout the hospital, supports the feasibility of adoption of a CDSS in their current workplace.

If there is a digital device, the less it can be handled the better. Now if there is a storage space, then the handling will be less. And here there is a charger point on the bed in every bed. There should not be much difficulty for them.

Nurse from ICMH

Another nurse further emphasized that providers would generally be welcoming to a CDSS that could improve care for children with sepsis.

Often, we may not notice a child's condition has deteriorated until we check again because we are also attending to other children. A device that detects when a child's condition worsens due to sepsis, just by being in contact with their body, would help us give that child immediate attention. This would improve the ability to monitor and respond to the needs of septic children more effectively.

Nurse from ICMH

In addition, providers reported that a CDSS could help improve workflow since other staff could use the devices and report updates to the physicians. The devices were also seen as valuable for providing rapid, continuous, and remote updates on patients’ conditions, helping with early detection and management, and enabling the monitoring of multiple children at once. One physician noted:

...actually, I’m going to be the only doctor working. When my mobile shows that, it is definitely very easy for me, comfortable for me, that I can see it in one mobile. But if multiple devices are connected, like connected with my sisters [nurses], if for some reason I went to the washroom or I went to pray or to a meeting or to dinner or lunch, then for any reason if the sister realizes that the patient is getting worse, she can immediately inform me. Then, I could also follow-up with the sisters, then there would be no restrictions on the follow-up of the children in my absence.

Physician from icddr,b

### Feasibility of CDSS Implementation

Both nurses and physicians considered the CDSS feasible for implementation into their sepsis treatment protocol. They specifically highlighted the potential for the monitoring system to improve the quality of care, save time for providers, and improve workflow efficiency by reducing the number of required staff. It was also noted that the CDSS would alleviate nurses’ anxiety related to patient monitoring by providing a reliable way to easily monitor patients’ stability. HCPs also expressed that its wireless capacity supports integration by reducing entanglement of wires from traditional monitoring devices and lowering the risk of infection spread due to the remote aspect of wireless, wearable biosensor devices.

Yes, it will be convenient for work. When the doctor comes for rounds, they can start treatment immediately upon hearing from me. I'll connect the device via my phone and inform them about the child’s condition. The doctor won't need to spend much time monitoring because they'll see what they need to see, and based on that, they can take their next steps.

Nurse from KGH

It’s good. We won’t have to touch the baby that much. We can see everything in the monitor, not having to touch the baby. Less touches mean less chances of infection.

Nurse from KGH

It will be very beneficial for all of us who work in the children's department, because as a nurse when I get updates on these things for a baby... as soon as I put on the monitor and know the baby's total (vitals), mentally I will be relieved that this baby is fine.

Nurse from KGH

However, there were reservations about its feasibility for initial integration with existing monitoring equipment and trust in its functionality, for which participants stressed the importance of training.

There will be no issues from doctor, nurse, or patient side. But currently I’m not sure if there will be any technical problem. I’m not sure if it will be well functioning all the time…I mean we will have to check if there’s any technical drawback.

Physician from KGH

### Preferences for CDS and Alerts

Both physicians and nurses expressed that the mHealth CDS tool would be most useful if it provided alerts for abnormal vital signs. Clinicians desired the mHealth CDS tool to accurately and reliably collect standard vital signs (such as heart rate, respiratory rate, blood pressure, and oxygen saturation), and have adjustable alarm parameters. Many favored audio (ringtone) or visual (light) alarms to alert about changes in captured vital signs. One provider cautioned that alarms should not be continuous or excessive to avoid alarm fatigue and disturbances to the patient.

Having an alarm system is beneficial. When the heart rate or saturation decreases, the alarm alerts us, allowing me to promptly inform the doctor upon arrival. The doctor assesses the patient, confirming if the baby is fine. This process is beneficial for us.

Nurse from ICMH

Continuous alarm can also disturb the child in both day and night. It can also create problems for the duty doctor or nurse, so the alarm should only beep during the deterioration.

Physician from KGH

### Preferences for CDSS Functionality

Both nurses and physicians at multiple hospitals discussed the advantages of having a central monitoring system that allows multiple patient vitals to be displayed on a single monitor. A feature to connect multiple patient devices to a single mobile device or central system was noted to be particularly helpful in settings with limited staff resources. As one physician expressed:

If multiple devices can be connected to one phone, it will be much more helpful because normally I am the only doctor. I do not have 14 doctors, who will be connected for 14 patients.

Physician from icddr,b

Another physician from icddr,b described the advantage of being able to access patient data remotely on a mobile device, explaining:

It’s a small device. I can follow up on my mobile. I can’t go to every child's bed to see the monitors. I can follow up with my device while sitting... Every child's follow-up is in front of my eyes. I can notice while doing any work, so I can catch any change. It will be very easy for me.

Physician from icddr,b

Additionally, nurses and physicians underscored the need for additional features, such as blood pressure measurement, and suggested that the CDSS should also have the capacity to include laboratory biomarker monitoring and notifications such as blood count, bilirubin, glucose, and additional electrolyte levels, enabling them to monitor other parameters that could indicate sepsis. Nurses recommended the ability for health care staff to set alarm parameters and silence alarms or mark alarms as seen. Physicians also mentioned that the CDSS should be able to detect seizure activity and apnea, as well as assess capillary refill time and hydration status.

One is of course heart rate, [then]saturation, respiratory rate, and if there is temperature then it is very good. Sepsis children develop hypothermia more often, [so] these four are mandatory.

Physician from ICMH

The alarm must [have] respiratory rate, heart rate and saturation. Saturation is not there for now. [Also] temperature, of course, there should be an alarm if there is hypothermia.

Physician from ICMH

Physicians highlighted the importance of durability and data storage capacity, recommending that patient information could be archived for later review if needed.

Both nurses and physicians preferred a wireless wearable biosensor device that could operate without having to be plugged into an electrical outlet and could use alternative power sources, such as solar power, to ensure continuous monitoring. Nurses specifically noted the need for a small, easily cleaned, skin-friendly wearable biosensor device that is appropriate for infant and child use and that could be applied to multiple areas of the body without causing skin irritation in infants with delicate skin.

The wireless patch, on the other hand, is very nice, modern, and comfortable to use. Cables in monitors cause a lot of trouble, especially when a bubble CPAP line gets twisted with the monitor cables, [it] creates a clumsy and tangled situation. From a hygiene point of view, the wireless patch is much better. Although we wash the wires with alcohol and reuse them for patients, the wireless option is more hygienic and avoids the hassle of dealing with cables.

Nurse from icddr,b

The thing is that this should be skin friendly. That is, the patch and the placement that I am opening should be skin friendly, soft. The children should not feel [that the device is] too hard, and there should not be any irritation. This is my first point for comfortability because what we often see is skin rashes [on children] when we put monitor cords on them. They get red, they get irritated, [or] children get hurt. That's why being skin friendly is the most important thing for this.

Physician from icddr,b

## Discussion

### Primary Findings

The findings of this study underscore the potential impact and high perceived feasibility and acceptability of a CDSS (wearable biosensor device and mobile CDS tool) for pediatric sepsis management in low-resource settings, such as Bangladesh. The nurse and physician participants in this study expressed overall high optimism regarding the potential positive impact that a CDSS could have in alleviating the challenges they face in sepsis management. By providing real-time continuous vital sign monitoring, a major current limitation due to resource constraints, participants perceived that novel CDSS technologies would allow them to prioritize high-risk patients and initiate timely interventions, thereby reducing mortality rates. Additionally, participants noted that a CDSS could potentially reduce staff workloads, particularly during times of high patient volumes and in clinical settings with health care worker shortages, by reducing the need for frequent manual assessments and enhancing workflow efficiency.

Participants often understood the real-time monitoring and predictive risk scoring capabilities of this CDSS as a single function, making it important to clarify the distinction and provide dedicated training on functionality during implementation efforts. The providers acknowledged that real-time monitoring of vital signs can enhance clinical decision-making by allowing providers to prioritize deteriorating patients. Additionally, some participants identified the value of a predictive algorithm that can provide estimates of mortality risk and proactively alert providers to patients who have a high risk. Prior work on digital CDS tools in Bangladesh has demonstrated that prediction models derived from clinical features can outperform the current standard guidelines for clinical management in both accuracy and reliability [24]. Using mobile apps to deliver real-time decision support illustrates how data-driven risk assessments can be feasibly implemented by clinicians in a low-resource setting.

While feedback on the utility of digital technology for pediatric sepsis was largely positive, HCPs identified several essential conditions for successful integration of such tools in Bangladesh and other LMIC contexts. They emphasized that, given the existing gaps in sepsis protocol adherence and technology use, targeted training on device functionality and the importance of pediatric sepsis monitoring would be crucial. Nurses and physicians also recommended adapting the device to include features tailored to their specific needs, workflows, and infrastructure availability. They indicated that having centralized monitoring devices capable of monitoring multiple patients simultaneously, introducing alert systems customizable according to clinician preferences and patient needs, and having comprehensive data storage would enhance usability and acceptability. Concerns were raised about the device’s affordability and durability, with clinicians stressing the importance of making it accessible, especially in government-funded hospitals that serve low-income patients.

Participants were overall highly engaged by the prospect of digital technology for pediatric sepsis care and thought creatively about solutions to overcome potential implementation barriers, such as suggesting solar panels to ensure an electrical supply to charge devices in settings where electricity is not continuously guaranteed. Additionally, participants mentioned that other priority features should be included in future wearable-based monitoring systems, such as electrolyte, glucose, and laboratory biomarker monitoring. While this is not feasible in the prototype design of the system this research team is developing, wearable biosensor devices capable of electrolyte monitoring through sweat and wearable ultrasound devices are rapidly expanding the possibilities available for monitoring through novel wearable technologies [25].

### Comparison to Prior Work

Our findings support themes identified in previous research on CDS tools, particularly the importance of flexibility and customization. One study highlighted the need to tailor CDS tools for pediatric sepsis to varying levels of medical training as well as local differences in culture, language, and resources [10]. Similarly, the participants in our study underscored the value of user customization and mentioned that the ability to tailor alert parameters at the level of individual providers would enhance workflow with the tool. The importance of administrative support to integrate novel digital technologies into existing systems was also a key finding. Other considerations for implementation include having offline functionality or solar-powered battery options to mitigate internet connectivity challenges and having a simple user interface.

### Limitations

This study has several limitations that must be considered when interpreting the findings. First, apart from 1 participant, the health care workers who participated in the study did not have direct experience using wearable biosensor devices. As such, their perceptions and feedback may differ from those of providers who have hands-on experience with wearable biosensor devices and newer digital technologies.

Second, the study did not include other types of stakeholders, such as policy makers, hospital administrators, or other clinical personnel, whose perspectives and operational insights could provide a more comprehensive understanding of the device’s potential impact on workflows and resource use.

Third, the hospitals included in the study are not representative of all health care facilities in Bangladesh, particularly those in more rural or remote areas where resource constraints and workflows may differ significantly. In LMIC settings, it is essential for any CDSS to function without relying on continuous power sources or internet connection and have the capacity to store data offline when necessary. Having a rechargeable and reusable wearable biosensor device and considering alternative power supplies, such as solar power, to ensure charging sources for mobile devices can improve the feasibility of implementing this system in low-resource settings. The generalizability of the findings to hospitals operating in these settings is limited, and the findings are likely most relevant to relatively high-resourced hospitals in urban regions of other LMICs. This research team is currently planning to conduct further preimplementation research in public, rural, and hard-to-reach hospitals in Bangladesh to gain a better understanding of implementation barriers and facilitators in more diverse settings in the country.

Lastly, the findings may not be widely applicable beyond the specific context of this study, given the unique organizational and cultural characteristics of the hospitals included. Broader applicability would require further validation in diverse health care settings, both within Bangladesh and in other low-resource contexts.

### Future Directions

Studies to evaluate the clinical impact of digital CDS tools in LMICs are greatly needed to better understand the true value that these tools may provide over the standard of care and traditional clinical management guidelines and tools. More research is also needed regarding how clinicians, particularly in LMICs, understand clinical prediction models and what factors may influence their trust or mistrust of prediction models, particularly in an age of increasing prevalence of machine learning and artificial intelligence in clinical care.

Notably, there appeared to be challenges among nurse participants in differentiating the functions of vital sign monitoring from clinical risk prediction in the proposed tool demonstrated to them. This likely reflects a lack of familiarity with other CDS tools and prediction models in clinical care, as well as difficulty communicating abstract concepts, such as risk prediction, among general practice nurses with minimal or no formal statistical exposure in their training. Further research should explore how best to educate nurses and other health care workers in low-resource settings about clinical prediction models in clear and understandable terms in order to build confidence and trust in using CDSSs and prediction models for potentially improving the care of patients and supporting their clinical decision-making. It is also possible that these issues were compounded by participant fatigue, as the questions addressing this distinction were posed toward the end of the interviews.

Efforts to ensure financial accessibility were identified as critical to any plans to scale up the use of similar technologies in Bangladesh. This includes exploring sustainable funding models, such as subsidies, public-private partnerships, and integration into national health systems, to reduce cost barriers for health care facilities and patients. Without such measures, the adoption and widespread use of these tools may remain limited, undermining their potential to improve pediatric sepsis outcomes. Robust training programs for HCPs were also identified as crucial for the scale-up of these technologies, as they help build familiarity and confidence in using these technologies while addressing gaps in understanding predictive functions and decision-support capabilities.

Implementation science frameworks offer a valuable structure for guiding the design and evaluation of interventions. As this study represents the preimplementation phase, our goal was to first explore and characterize context-specific barriers and facilitators. Our future research will use the Consolidated Framework for Implementation Research 2.0.

### Conclusion

While wearable biosensor devices and CDS tools were viewed as a promising solution for improving pediatric sepsis management in Bangladesh and other LMICs, careful planning and a nuanced understanding of the local context will be essential for the successful implementation of these technologies in the future. These tools have the potential to bridge critical gaps in early detection and timely intervention for pediatric sepsis, but their success hinges on addressing key factors, including adapting digital technologies to suit local clinical contexts and needs to ensure usability and effectiveness; customization of CDS features by end users; ensuring adequate infrastructure, particularly electrical supply; and providing profession-specific training on how the tools can be best used to support health care workers and patients.

Finally, engaging a broader range of stakeholders, including nurses, technicians, hospital administrators, and policy makers, will be critical in identifying and mitigating potential operational and systemic challenges during implementation. By adopting a holistic and inclusive approach, this model could inform the integration of similar digital health technologies across other low-resource settings, paving the way for scalable and sustainable advancements in global health.

## Data Availability

The datasets generated or analyzed during this study are available from the corresponding author on reasonable request.

## Appendix

Multimedia Appendix 1 SRQR checklist.

Multimedia Appendix 2 Participant demographic questionnaire.

Multimedia Appendix 3 Interview guide.

## Abbreviations

CDS: clinical decision support
CDSS: clinical decision support system
HCP: health care provider
icddr,b: International Centre for Diarrhoeal Disease Research, Bangladesh
ICMH: Institute of Child and Mother Health
IRB: Institutional Review Board
KGH: Kushtia General Hospital
LMIC: low- and middle-income country
mHealth: mobile health
