# Supplementary figures and images for: Health Care Providers’ Perspectives of Clinical Decision Support Tools for Pediatric Sepsis in Bangladesh: Qualitative Study
Source: JMIR Form Res. 2025 Sep 26;9:e73451. doi: 10.2196/73451 (PMC12514408; doi:10.2196/73451)

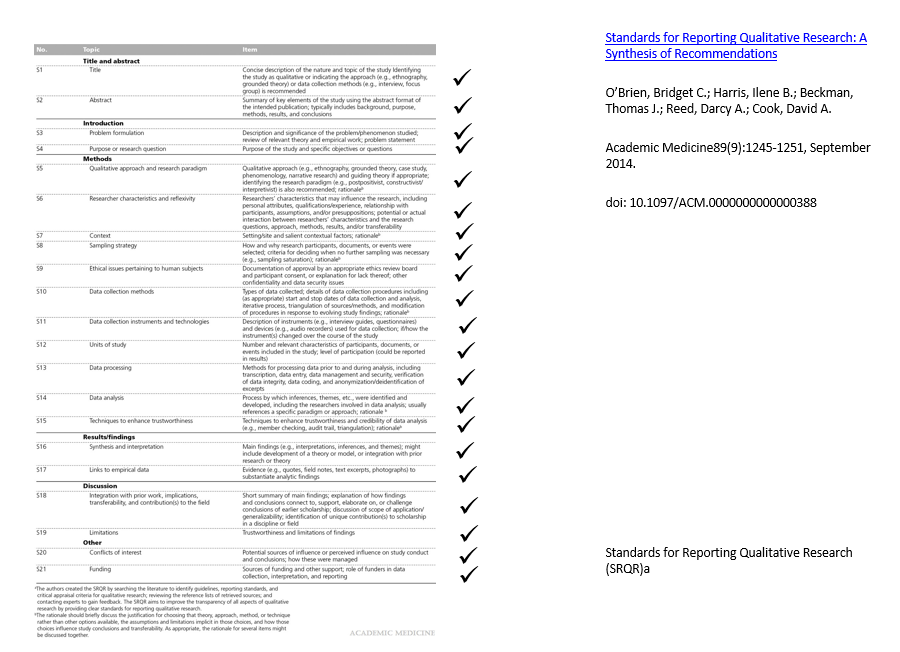

Supplement: Multimedia Appendix 1 [file formative_v9i1e73451_app1.png]
