# Supplementary material for: Health Care Providers’ Perspectives of Clinical Decision Support Tools for Pediatric Sepsis in Bangladesh: Qualitative Study
Source: JMIR Form Res. 2025 Sep 26;9:e73451. doi: 10.2196/73451 (PMC12514408; doi:10.2196/73451)
Supplement: Multimedia Appendix 2 [file formative_v9i1e73451_app2.docx]

**REMEDIES Qualitative Study:**

**Participant Questionnaire**

The researchers who are running this study collect descriptive information to describe the people who are asked to participate in the study. Please check the box next to the response that best matches your opinion.

AGE (years)
 18-24

 25-34

 35-44

 45-54

 55+

GENDER

 Female

 Male

EDUCATION
 Diploma

 Bachelor’s degree

 College Degree

 Master’s Degree

 Doctorate

WORKING STATUS

 Full Time

 Part Time

CURRENT POSITION

 Physician

 Nurse

NUMBER OF YEARS OF EXPERIENCE AT CURRENT POSITION

___ Years

NUMBER OF YEARS OF EXPERIENCE TREATING CHILDREN WITH SEPSIS

___ Years

APPROXIMATE NUMBER OF CHILDREN WITH SEPSIS TREATED PER MONTH

 0-1

 2-5

 6-10

 11-20

 >20
