# Supplementary material for: Health Care Providers’ Perspectives of Clinical Decision Support Tools for Pediatric Sepsis in Bangladesh: Qualitative Study
Source: JMIR Form Res. 2025 Sep 26;9:e73451. doi: 10.2196/73451 (PMC12514408; doi:10.2196/73451)
Supplement: Multimedia Appendix 3 [file formative_v9i1e73451_app3.pdf]

QUALITATIVE INTERVIEW GUIDE – LONG FORM

The intent of this research is to gather feedback to support the creation of a new mobile health tool to help clinicians better monitor and care for children with sepsis in Bangladesh. The tool will consist of a wearable device and mobile "app" to 1) provide continuous vital sign monitoring, 2) alert health care providers of signs of patient deterioration, and 3) estimate the risk of severe sepsis and death. Focus groups will ask about current app and digital device use and then will seek feedback about the development of the new REMEDIES tool to help clinicians monitor and better care for patients.

Interviews will be facilitated by the REMEDIES icddr,b-based research staff and clinicians, as well as Brown University-based research team members. Interviews will be conducted in Bangla and/or English, whichever is most suitable to the participants. Interviews will be audio recorded and then translated and transcribed prior to analysis. In addition, a debrief will be written by the facilitator/notetaker after each interview and reviewed with the project team. Debriefs are used to provide facilitation feedback and assess data gathered, including saturation.

This agenda is intended to guide facilitators through the key content areas, ensuring that the same content is discussed in each interview. While the agenda is used to guide the discussion, it is not a rigid script that will be adhered to verbatim. This ensures that the facilitators gather data on the same topics, while also allowing them the flexibility to adapt and clarify questions to suit the needs of different individuals. Similarly, questions need not be asked in this particular order. Rather, the facilitator will adapt the conversation as needed according to the interview, pursuing both the a priori research topics as well as any emergent relevant themes that evolve from the discussion. Intent statements describe the data each agenda section is designed to collect.

A. Welcome and Introduction [approx. 5 minutes]:

Intent: *The goal of Section A is to introduce the facilitator and co-facilitators, welcome participants and explain the purpose of the interviews.*

1. Greetings and thank participants for joining the interview.
   a. Offer water/tea and refreshments.
   b. Introduce facilitator(s), note taker, and any other researchers who are present.
   c. Informal conversation to create rapport with participants. (Can begin prior to/during informed consent process. How is their work? Their hometown? Weather, traffic, food, sport, etc.)
   d. Conduct informed consent process, sign consent form.
   e. Ask participant to complete the study questionnaire using pen.

2. Explain that we work with a research team that is developing a new mobile health tool, which will help doctors and nurses better monitor and care for children with sepsis.
3. We researchers are here to learn about your experiences and opinions. There are no right or wrong answers and you never have to answer any question you don't want to.
4. The interview will be audio recorded. We will be making a transcript and an English translation of this discussion. When we do that, we remove your name and other things that identify you or the patients/colleagues you work with. You will only be identified with your number. We keep your comments confidential and the audio recording of this interview is kept on a secure server and deleted at the end of the study.
5. At the end of the session, we will reimburse you for your time with some honorarium.
6. Please speak a bit loudly, so your voice is clear on the recorder.
7. Request that participants silence their phones.
   a. "We greatly value your input and would like your uninterrupted attention and ask for you to silence your phone for the interview."
8. We expect this interview to take about 1 hour, is that timing ok with you?
9. Ask participant if they have any questions, answer questions (if any), then turn on the audio recorders.

B. Information about treating children with sepsis:

***Intent*:** *The intent of this section is to understand the participants' experiences and current practices when caring for children with sepsis, including monitoring and making decisions regarding treatments. This will be used to later transition to talking about how the tool could help clinicians in these duties.*

1. Describe your role in your workplace.
   a. Your current role? Any previous roles?
2. Describe your experience identifying and treating children with sepsis (life-threatening infection).
   a. Explain the current practice at your hospital for caring for children with sepsis.
      i. Who is involved in patient management?
      ii. Describe any clinical tools, protocols or guidelines, if any, that are used in treating children with sepsis.
   b. Tell us about your most recent experience caring for a child with sepsis.
   c. How are decisions made regarding how to treat children with sepsis at your hospital?
      i. What are the most important actions to take when caring for a child with sepsis?
3. Explain how children with sepsis are monitored at your hospital.
   a. How are clinicians made aware of a child who is deteriorating due to sepsis?
      i. Tell me about the current practice for monitoring vital signs.
      ii. Tell me about the current practice for monitoring for signs of deterioration.
   b. What are the most important signs of clinical deterioration that you would like to know about?
      i. How is this information collected and recorded?
      ii. What do "danger signs" / "red flags" / "warning signs" mean?
   c. What measures are taken after a child with deterioration is identified?
   d. What are the some of the challenges that clinicians face in monitoring children with sepsis?
4. Describe some of the challenges that clinicians face in caring for children with sepsis.
5. Describe some of the things that help clinicians care for children with sepsis.
6. How do you feel when you are caring for children with sepsis?
   a. Reminder: Addressing issues of confidence, comfort, mental state, emotions, etc.

C. Understanding current and future digital tool use:

***Intent*:** *We want to learn what digital tools participants use in daily life and at work. This will serve as an introduction and provide information about participants' familiarity with technology, especially the use of digital health tools and wearable devices to inform the creation of our tool.*

"Thank you so much for sharing your experiences. We are interested in helping clinicians like you in your duties using a new digital tool to care for children with sepsis. So we want to understand your experience with digital tools."

1. Describe any digital devices you are aware of or use in your day-to-day life or at work.
   a. Computers? Mobile phones? Electronic health records?
   b. Why, when, how?
   c. What apps do you use?
   d. What is your opinion of digital devices?
2. Describe any wearable devices you are aware of or use.
   a. Smart watch, Fitbit, pulse oximeter, Bluetooth headset, Holter monitor, continuous tocogram (CTG), etc.?
   b. What about wearable devices for health purposes?

3. What do you think about the use of digital devices when caring for children with sepsis?
    a. Who should use them? How should they be used?
4. Describe barriers and facilitators to introducing digital devices at your workplace.
    a. How can these barriers be addressed?

D. Demonstration of tool

Short visual presentation of the tool in person with a mannequin to show placement of the device as well as mobile phone with test version of the phone application.

E. Feedback on the Tool

Intent: *The intent of this section is to get feedback on the participants' opinion on the example of the tool that was demonstrated, as well as understand their perspective on what it would be like to implement this tool in real life. This will help the research team understand issues that may impact future implementation that need to be addressed.*

(Transition: "*Now that you've learned about this tool that we're developing, we would value your feedback, both positive and negative. There are no right or wrong answers.")*

1. What do you think it would be like to use this tool when caring for children with sepsis?
    a. What do you see as the pros and cons?
        i. What do you like about it? What don't you like?
    b. How would your colleagues feel about using this tool? How would patients feel?
    c. Are there certain patients with whom it would be difficult to use?
        i. How about for patients with other conditions (other than sepsis)?
2. In order for people to use this tool, what should be considered?
    a. Cost-effectiveness? Durability? Reliability of vital signs? Adaptability? Sustainability? Training and skills? Need for super-users? Data privacy and ethical issues? Infrastructural issues?
    b. What challenges might there be to implement this tool at your workplace?
        i. Any circumstances when it may be difficult to use the tool?
            1. How can we make the tool usable in challenging situations?
        ii. What challenges might there be at other hospitals you have worked at?
    c. What are some things that would help with using this tool at your workplace?
    a. What practical conditions should be considered when using this tool?
        i. Electricity, internet connectivity, humidity and temperature, physical layout of your workplace?
2. How would you feel using the tool?

F. Use of the Tool for Monitoring and Clinical Decision Support

Intent: *The intent of this section is to understand participants' preferences, advice, and recommendations for creating the tool, which will allow continuous patient monitoring as well as detect patient deterioration to help clinicians make decisions.*

(Transition: "*Thank you for that information. We would now like to know your opinions to help develop this tool to detect patient deterioration and make decisions. Tools like this may be able to provide alerts.")*

1. What are the most important things you would like an alert to be generated for?
    a. Abnormal vital signs?
        i. Fast or slow heart rate, respiratory rate, high/low temperature?

    b. What else would be important to measure using wearable devices?
        i. Oxygen saturation, blood pressure
        ii. Irregular heart rate / ECG abnormalities?
2. If this tool could alert you to patient deterioration, how would you prefer to be alerted?
    a. Notification on screen, phone rings/vibrates, text message sent, phone call, etc.?
    b. When and who gets alerted?
    c. What are the best ways to avoid “alarm fatigue”?
2. How could this tool be used to make clinical decisions?
    a. To make treatment decisions (IV fluids, vasopressors, other medications)? Ordering laboratory investigations? Counseling families? Advice from consultants?
    b. Decide whether the child needs referral to another health facility?
3. If this tool could estimate the patient’s risk of severe sepsis or death, how would you use this information?
    a. What would be the impact of this tool on patient care?
4. Do you have any other ideas for how we should develop this tool?

G. Conclusion. [approx. 5 minutes]:

***Intent*:** *Wrap up the interview, thank the participants, etc.*

1. Is there anything else we need to know? Anything important that we didn’t ask about?
2. Thank you for your time.
3. Reimbursement or honorarium.

POST INTERVIEW: Fill out debrief form. Save and download audio files. Make recording available for transcription.
